# Anodal transcranial direct current stimulation to the cerebellum improves handwriting and cyclic drawing kinematics in focal hand dystonia

**DOI:** 10.3389/fnhum.2015.00286

**Published:** 2015-05-18

**Authors:** Lynley V. Bradnam, Lynton J. Graetz, Michelle N. McDonnell, Michael C. Ridding

**Affiliations:** ^1^Discipline of Physiotherapy, Graduate School of Health, University of TechnologySydney, NSW, Australia; ^2^Discipline of Physiotherapy, School of Health Sciences, Flinders UniversityAdelaide, SA, Australia; ^3^Sansom Institute for Health Research, School of Health Sciences, University of South AustraliaAdelaide, SA, Australia; ^4^Robinson Institute, School of Paediatrics and Reproductive Health, University of AdelaideAdelaide, SA, Australia

**Keywords:** cerebellum, transcranial direct current stimulation, cerebellar-brain inhibition, focal hand dystonia, handwriting, kinematics

## Abstract

There is increasing evidence that the cerebellum has a role in the pathophysiology of primary focal hand dystonia and might provide an intervention target for non-invasive brain stimulation to improve function of the affected hand. The primary objective of this study was to determine if cerebellar transcranial direct current stimulation (tDCS) improves handwriting and cyclic drawing kinematics in people with hand dystonia, by reducing cerebellar-brain inhibition (CBI) evoked by transcranial magnetic stimulation (TMS). Eight people with dystonia (5 writer’s dystonia, 3 musician’s dystonia) and eight age-matched controls completed the study and underwent cerebellar anodal, cathodal and sham tDCS in separate sessions. Dystonia severity was assessed using the Writer’s Cramp Rating Scale (WRCS) and the Arm Dystonia Disability Scale (ADDS). The kinematic measures that differentiated the groups were; mean stroke frequency during handwriting and fast cyclic drawing and average pen pressure during light cyclic drawing. TMS measures of cortical excitability were no different between people with FHD and controls. There was a moderate, negative relationship between TMS-evoked CBI at baseline and the WRCS in dystonia. Anodal cerebellar tDCS reduced handwriting mean stroke frequency and average pen pressure, and increased speed and reduced pen pressure during fast cyclic drawing. Kinematic measures were not associated with a decrease in CBI within an individual. In conclusion, cerebellar anodal tDCS appeared to improve kinematics of handwriting and circle drawing tasks; but the underlying neurophysiological mechanism remains uncertain. A study in a larger homogeneous population is needed to further investigate the possible therapeutic benefit of cerebellar tDCS in dystonia.

## Introduction

Focal or isolated dystonia is a neurological disorder where involuntary muscle contractions cause intermittent or sustained abnormal postures of an isolated body part, such as the hand (Fahn, 1984; Albanese et al., 2013). Focal hand dystonia (FHD) is a task-specific form of focal dystonia, common in writers and musicians. Despite intensive research efforts, the pathophysiology underlying FHD remains unclear. There is growing evidence that the cerebellum and basal ganglia are key components of an integrated brain network contributing to dystonia (Jinnah and Hess, 2006; Argyelan et al., 2009; Neychev et al., 2011; Standaert, 2011; Sadnicka et al., 2012; Bradnam and Barry, 2013; Filip et al., 2013; Prudente et al., 2013). The excitability of the cerebellar to primary motor cortex (M1) pathway can be probed in humans using paired-pulse transcranial magnetic stimulation (TMS), known as cerebellar-brain inhibition (CBI; Ugawa et al., 1995; Daskalakis et al., 2004; Koch et al., 2008). The cerebellum exercises tonic inhibition over M1 via the cerebellothalamocortical pathway for precise control of the hand during an active task (Kassavetis et al., 2011). It is possible that motor deficits experienced in neurological movement disorders such as dystonia, might partially arise from aberrant cerebellar modulation over M1. In support, CBI is reduced in FHD (Brighina et al., 2009), progressive supranuclear palsy and Parkinson’s disease (Carrillo et al., 2013; Brusa et al., 2014). The impact of a reduction of CBI on motor function remains unclear and more studies are needed to clarify exactly how cerebellar dysfunction demonstrated by CBI impairs movement in neurological disorders.

The evidence that the cerebellum may play a role in the pathophysiology of focal dystonia suggests there may be novel therapeutic opportunities, as the superficially located cerebellum is easily targeted with non-invasive brain stimulation (Grimaldi et al., 2014). There have been reports of small clinical improvements, associated with an increase in CBI, after cerebellar continuous theta-burst stimulation (TBS) in cervical dystonia (Koch et al., 2014) and intermittent TBS in progressive supranuclear palsy (Brusa et al., 2014). More specifically, Sadnicka and colleagues examined the effect of cerebellar transcranial direct current stimulation (tDCS) on motor cortical plasticity evoked by paired-associative stimulation in people with a specific form of FHD known as writing dystonia (Sadnicka et al., 2014). In contrast to the TBS studies, functional outcome measures of handwriting assessed by the Writer’s Cramp Rating Scale (WRCS; Wissel et al., 1996), time to complete a standardized sentence and self-rated improvement using a visual analogue scale, were unaffected by cerebellar tDCS (Sadnicka et al., 2014). However, handwriting kinematics was not assessed in that study. Kinematic analyses of handwriting and cyclic drawing are sensitive to change following interventions in people with FHD (Zeuner et al., 2007; Schabrun et al., 2009) and, therefore, are worthy of investigation in a study of cerebellar tDCS.

The aim of this exploratory study in a small number of FHD participants was to examine whether sensitive measures of hand function/neurophysiology provide evidence that cerebellar tDCS improves FHD. The first hypothesis was that CBI would be reduced in people with FHD, with concomitant deficits in handwriting and cyclic drawing. The second hypothesis was that anodal tDCS of the cerebellum would influence both CBI and handwriting and cyclic drawing in people with FHD.

## Experimental Procedures

### Participants

Eight people with FHD (5 writer’s cramp, 3 musician’s cramp) diagnosed by a neurologist were enrolled in the study (age range 37–80, 7 male). Participants were recruited from a research database and dystonia support network groups. Eight control participants (age range 44–83, 6 male) without neurological or musculoskeletal disorders of the upper limb or hand were recruited by advertisement from university staff where the study was conducted (Table 1). All control participants were right handed according to the Edinburgh Handedness Inventory (Oldfield, 1971). All but one participant in the dystonia group were right-handed, and all but one participant experienced dystonia in their dominant hand. While three FHD patients were diagnosed as having musicians dystonia, all three also experienced handwriting dystonia, as evidenced by their WCRS scores (Table 1). No FHD participants were undergoing rehabilitation for their dystonia at the time of the study, although three had completed a course of physiotherapy consisting of exercises and relaxation in the past 12 months with little benefit. None had ever been treated with botulinum toxin injections for their FHD. All participants provided written informed consent prior to the study and were screened for safety to undergo TMS and tDCS using a customized version of a standard tool (Rossi et al., 2009) by a medical doctor. The study was approved by the local ethics committee.

**Table 1:** **Characteristics of people with dystonia and controls**.

Dystonia Group							Control Group		
Gender	Age (yr)	Type	Time since onset (yr)	Handedness (EHI)	WCRS (0–30)	ADDS (0–100)	Gender	Age	Handedness (EHI)
M	70	WD	8	92	5.7	35.2	F	71	92
M	57	MD	6	88	3	82.1	M	59	92
M	63	WD	18	92	10	50.9	M	62	92
M	60	WD	17	25	8.7	50.9	M	62	83
M	80	MD	11	92	15.7	50.9	M	83	88
F	37	WD	3	92	7	54.2	F	44	92
M	50	MD	5	88 †	7	34.4	M	54	88
M	55	WD	4	92	4	50.9	M	55	88

*Abbreviations: WD, Writers Dystonia; MD, Musicians Dystonia; ADDS, Arm Dystonia Disability Scale (higher scores, less disability); WCRS, Writer’s Cramp Writing Scale (higher scores, greater impairment); EHI, Edinburgh handedness inventory. † This participant experienced dystonia in their non-dominant hand*.

### Experimental Design

Participants attended three experimental sessions, separated by at least 5 days. The sessions were identical apart from the tDCS intervention. First handwriting and cyclic drawing were recorded, followed by TMS measures of MEP amplitude and CBI. After TMS, cerebellar tDCS was delivered by a separate investigator, either as anodal, cathodal or sham tDCS, in random order across sessions. There was a five minute rest period after tDCS, before the post-intervention TMS measures were collected, followed by handwriting and cyclic drawing. Investigators conducting neurophysiological and behavioral testing and all participants were blinded to the tDCS intervention at each session. Dystonia severity was assessed using the Arm Dystonia Disability Scale (ADDS; Fahn, 1984). Writing impairment was graded using the WRCS (Wissel et al., 1996), by an independent assessor from video-recordings of participants writing on a digitizing board (see below). The trial was registered on the Australian New Zealand Clinical Trials Registry (ACTRN12612000339853).

### Electromyography

Surface EMG was recorded from the first dorsal interosseous (FDI) using 10 mm-diameter Ag/AgCl electrodes (Ambu, Ballerup, Denmark). Electrodes were placed over the muscle belly and the metacarpophalangeal joint. A 20 mm-diameter reference Ag/AgCl electrode was placed over the dorsum of the wrist (3M Health Care, St. Paul, MN, USA). Electromyography signals were sampled at 2000 Hz (CED 1401; Cambridge Electronic Design, Cambridge, UK), amplified by 1000 (CED 1902; Cambridge Electronic Design, Cambridge, UK), band-pass filtered (20–1000 Hz) and stored for offline analysis (Signal v5.09, Cambridge Electronic Design, Cambridge, UK).

### Transcranial Magnetic Stimulation

Single-pulse TMS was delivered with a figure of eight coil (70 mm wing diameter) (MagStim Co., Whitland, Dyfed, Wales), positioned over the primary motor cortex to induce a posterior to anterior directed current in the underlying brain. The “hotspot” for evoking contralateral motor-evoked potentials (MEPs) in FDI muscle of the most affected (dystonia) or dominant hand (control) was located and marked on the scalp. Resting motor threshold (RMT) was determined as the minimum stimulus intensity to elicit an MEP of at least 50 μV in at least four out of eight trials (Rossini et al., 1994). To assess corticomotor excitability, sixteen MEPs were evoked at 120% RMT in the relaxed FDI muscle. The protocol used to assess CBI was as follows. A test stimulus was delivered over M1 using a figure of eight coil (70 mm wing diameter), preceded by a conditioning stimulus applied over the lateral cerebellum 3 cm lateral and 1 cm inferior to the inion. The conditioning pulse was delivered by a second flat figure of eight TMS coil, positioned with the handle pointing superiorly (Koch et al., 2007, 2008; Carrillo et al., 2013). The interstimulus interval between magnetic pulses was 5 ms (Ugawa et al., 1995). Sixteen non-conditioned and 16 conditioned MEPs were evoked at random at a rate of 0.2 Hz to assess CBI. The intensity for the test and conditioning pulses were established in the following manner. The test stimulus intensity was set to that which evoked a MEP in FDI of approximately 50% of the maximum MEP response (50% MEP_MAX_). To establish 50% MEP_MAX_, the intensity producing the largest MEP (MEP_MAX_) in FDI was identified, and then lowered incrementally until a consistent 50% MEP_MAX_ response was observed. For the conditioning stimulus, TMS was applied over the cerebellum at the intensity required to achieve a MEP (RMT) in FDI.

### Handwriting and Cyclic Drawing Tasks

Handwriting and cyclic drawing tasks were performed with participants’ seated in a comfortable writing position in front of a pressure sensitive digitizing tablet (WACOM Intuos A4 oversize; Wacom Europe, Germany), connected to a personal computer. Using an inking and digitizing pen, participants were first asked to draw superimposed circles approximately 2 cm in diameter for a period of 10 s as quickly as possible and then to repeat the task using minimal pen pressure (Zeuner et al., 2007; Schabrun et al., 2009). Participants were also asked to write the sentence “Sheila collects shells” 10 times in their normal handwriting (Schabrun et al., 2009). Specialized software (MovAlyzeR 4.1, Neuroscript USA) was used to record handwriting and circle drawing kinematics at a sampling rate of 133 Hz with a resolution of 0.005 mm.

### Transcranial Direct Current Stimulation

Transcranial DCS was delivered at a constant current of 2 mA for 20 min via two 25 cm^2^ saline soaked sponge electrodes using a Chattanooga Ionto stimulator (Chattanooga Group, Hixon, TN, USA). One electrode was centered over the lateral cerebellum, 3 cm lateral and 1 cm inferior to the inion and the other positioned over the right buccinator muscle (Galea et al., 2009). Sham tDCS was applied using the same electrode configuration, but current intensity was ramped down to zero after 30 s (Gandiga et al., 2006).

### Data Analysis

Kinematic analysis was performed using customized software (MovAlyzeR 4.1, Neuroscript USA). For handwriting, the word “Sheila” from the last three completed sentences was extracted and visually checked. If the word was incomplete, the algorithm was adjusted for that individual and the extraction process repeated. Once this criterion was satisfied, the average pen pressure in pascals (APP) and mean stroke frequency (MSF, 1/average stroke duration per minute) were calculated. The same kinematic measures were calculated for the fast and light cyclic drawing tasks. Data were tested for normality using the Shapiro-Wilk test and data that did not meet assumptions of normality were linearly transformed. To compare the groups at baseline, APP and MSF were separately analyzed by a three CONDITION (anodal, cathodal, sham) repeated measures ANOVA (rmANOVA). Where (as expected at baseline) there was no main effect of CONDITION, data were collapsed for GROUP comparison using independent sample *t*-tests (two-tailed). The relationship between baseline variables (kinematics, neurophysiology and clinical tests) was examined using linear regression analysis for the dystonia group. To assess the effect of cerebellar tDCS, MSF and APP were analyzed using a two GROUP (FHD, control) by three CONDITION (anodal, cathodal, sham) by two TIME (pre, post) repeated measures ANOVA.

For neurophysiological data, motor-evoked potential amplitude was measured peak to peak (mV) and averaged for each individual at each time point. For CBI, MEPs were expressed as a ratio of conditioned to non-conditioned MEP amplitude, so that a ratio < 1 signified inhibition of M1 by cerebellar stimulation. Data were tested for normality using the Shapiro-Wilk test and all data conformed. Baseline measurements for MEPs and CBI were analyzed using a three CONDITION (anodal, cathodal, sham) rmANOVA. Where there was no main effect of CONDITION data were collapsed for GROUP comparison using an independent sample *t*-test (two-tailed). The effect of cerebellar tDCS on MEPs, CBI and non-conditioned MEPs evoked for CBI were tested using a two GROUP (FHD, control) by three CONDITION (anodal, cathodal, sham) by two TIME (pre, post) rmANOVA. For comparison of kinematic and neurophysiological assessments, data were expressed as the difference between pre and post stimulation (post—pre, ∆MSF, ∆APP). SPSS software (Version 20, SPSS Inc, Chicago, USA) was used for all statistical analysis, with the level of significance set to *P* < 0.05. Mauchly’s test examined data for sphericity and the Greenhouse-Geisser correction used where data were non-spherical. Data are presented as the group mean ± standard error.

## Results

Participant demographics are presented in Table 1. There were no adverse effects of tDCS reported by participants in either group.

### Baseline Measures

**Figure 1:**
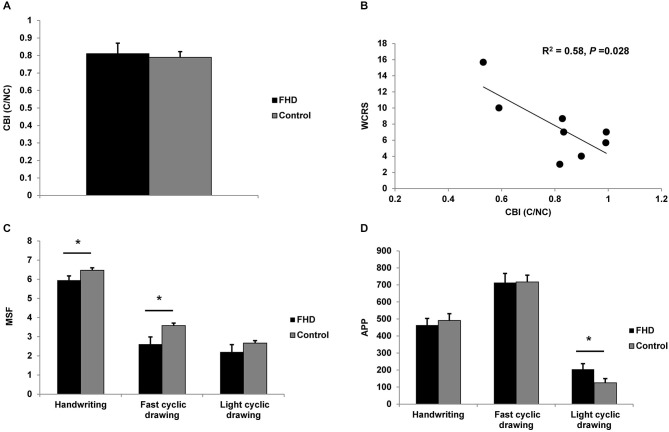
**Comparison of groups at baseline. (A)** cerebellar-brain inhibition (CBI). There was no difference in CBI between groups. **(B)** Moderate negative correlation between WCRS and CBI at baseline, showing greater CBI is associated with higher (worse) WCRS scores. **(C)** Mean stroke frequency (MSF) for the three tasks. The dystonia group were slower than the control group for handwriting and fast cyclic drawing. A similar pattern was observed for light cyclic drawing. **(D)** Average pen pressure (APP) for the three tasks. There was a difference between groups only for light cyclic drawing. Significance at *P* < 0.05 is signified by *.

Analysis of neurophysiological data revealed no main effect of CONDITION on MEP amplitudes, CBI and non-conditioned MEPs evoked during CBI (all *P* > 0. 31). Data were collapsed across CONDITION for GROUP comparison by independent sample *t*-tests. Results are summarized in Table 2. There was no difference between groups (all *P* > 0.15). Therefore, our *a priori* hypothesis that CBI will be reduced in dystonia was proven incorrect (Figure 1A). There was a moderate, negative relationship between CBI at baseline and the WRCS (*R*^2^ = 0.58, *P* = 0.028) for people with FHD (Figure 1B). There was no relationship between CBI and the ADDs or between CBI and any kinematic measure at baseline (all *P* > 0.38). For kinematic data there were no significant effects of CONDITION (all *P* > 0.22) and data were collapsed for comparison of GROUP. There was a GROUP difference for MSF during the fast cyclic drawing and handwriting tasks (both *P* < 0.05). The dystonia group performed the task more slowly than controls. There was no difference between groups for MSF during light cyclic drawing (*P* = 0.27), although the dystonia group appeared slower (Figure 1C). The APP during light cyclic drawing was higher in the dystonia than the control group (*P* = 0.037) but was no different for fast cyclic drawing or handwriting (both *P* > 0.6) (Figure 1D).

**Table 2:** **Comparison of groups at baseline from data averaged across the three sessions**.

	Control group	Dystonia group	*P*-value
MSF—handwriting (1/average stroke duration per minute)	6.5 ± 0.13	5.9 ± 0.23	0.049*
MSF—fast cyclic drawing (1/average stroke duration per minute)	3.6 ± 0.40	2.6 ± 0.66	0.049*
MSF—light cyclic drawing (1/average stroke duration per minute)	2.7 ± 0.32	2.21 ± 0.67	0.27
APP—handwriting (Pa)	451 ± 40	466 ± 40	0.63
APP—fast cyclic drawing (Pa)	680 ± 64	713 ± 85	0.62
APP—light cyclic drawing (Pa)	121 ± 23	207 ± 33	0.037*
MEP amplitude (mV)	0.75 ± 0.15	0.86 ± 0.13	0.79
CBI (C/NC)	0.79 ± 0.33	0.81 ± 0.04	0.69
NC MEPs (mV)	1.61 ± 0.21	1.86 ± 0.32	0.53
rmsEMG (mV)	0.008 ± 0.001	0.009 ± 0.001	0.32

Significance at P < 0.05 is signified by *. MSF, Mean Stroke Frequency; APP, average pen pressure; Pa, Pascal; MEP, motor-evoked potential; CBI, cerebellar-brain inhibition; C, conditioned; NC, non-conditioned; mV, millivolt; rmsEMG, root mean square electromyography.

### Effects of Cerebellar tDCS

**Figure 2:**
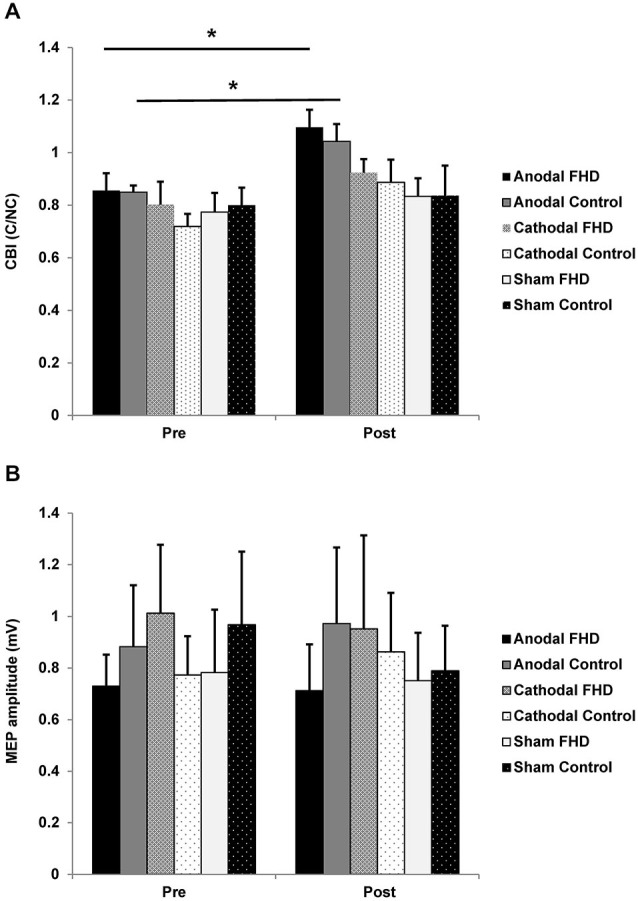
**Effect of transcranial direct current stimulation (tDCS) on cortical neurophysiology. (A)** CBI. There was an increase in CBI in both groups following anodal tDCS. **(B)** There was no change in MEP amplitude in either group. Significance at *P* < 0.05 is signified by *.

For CBI there was a main effect of CONDITION (*F*_2,14_ = 4.61, *P* = 0.044) and a main effect of TIME (*F*_1,7_ = 25.27, *P* = 0.002). *Post hoc* *t*-tests revealed a decrease in CBI in both groups following anodal tDCS (FHD Pre 0.86 ± 0.07, Post 1.1 ± 0.07, *P* = 0.003); control Pre 0.85 ± 0.03, Post 1.04 ± 0.07, *P* = 0.007). There was no main effect of group or any interactions (all *P* > 0.12) (Figure 2A). There was no change in non-conditioned MEPs evoked during CBI for either group (all *P* > 0.22); meaning conditions evoking CBI were consistent. For MEP amplitude there were no main effects or an interaction (all *P* > 0.12) (Figure 2B). There was no relationship between WRCS or ADDs and ∆CBI after anodal tDCS in the dystonia group (both *P* > 0.39). There was no relationship between ∆CBI and the change in any kinematic variable following anodal tDCS for either group (all *P* > 0.14).

**Figure 3:**
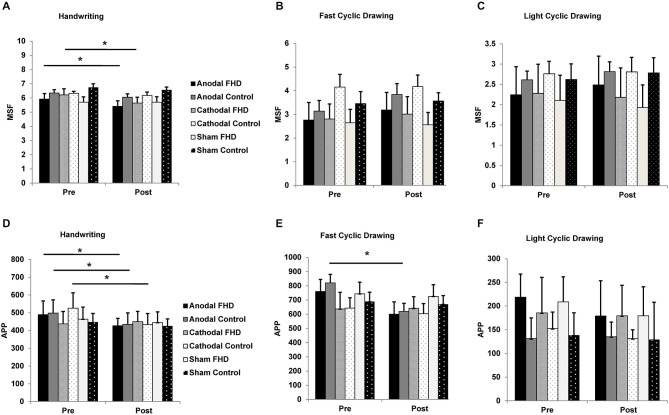
**Effect of tDCS on handwriting and cyclic drawing kinematics. (A)** Mean stroke frequency during handwriting was further reduced in the dystonia group by anodal and cathodal tDCS. **(B)** There was a strong trend for an increase in mean stroke frequency during fast cyclic drawing in both groups following anodal tDCS. **(C)** Mean stroke frequency in the light cyclic drawing task was unchanged by tDCS for either group. **(D)** Average pen pressure during handwriting was reduced by anodal tDCS in both groups and by cathodal tDCS in the control group. **(E)** Average pen pressure was reduced during fast cyclic drawing in both groups, although only the control group reached statistical significance. **(F)** Average pen pressure in the light cyclic drawing task was unchanged by tDCS. Significance at *P* < 0.05 is signified by *.

For handwriting MSF, there was a main effect of TIME (*F*_1,7_ = 26.46, *P* = 0.001) and a GROUP by CONDITION interaction (*F*_2,14_ = 6.31, *P* = 0.019) and no other main effects or interactions (all *P* > 0.18). Paired-sample *t*-tests revealed a reduction in MSF following anodal (pre 5.9 ± 0.39, post 5.4 ± 0.39, *P* = 0.015) and cathodal (pre 6.2 ± 0.44, post 5.6 ± 0.42, *P* = 0.033) cerebellar tDCS in the dystonia group only. There was no effect of sham tDCS in the dystonia group (*P* = 0.99). There was no change in the control group for any condition (all *P* > 0.83), although the pattern of results were consistent with the dystonia group (Figure 3A). There were no main effects or interactions for MSF in the fast cyclic drawing task, although there was a strong trend for a main effect of CONDITION (all *P* > 0.07), likely explained by an increase in MSF following anodal tDCS in both groups (Figure 3B). For MSF in the light circle drawing task there were no main effects or interactions (all *P* > 0.22, Figure 3C).

For handwriting APP there was a main effect of CONDITION (*F*_2,14_ = 4.87, *P* = 0.044) and a TIME by GROUP interaction (*F*_1,7_ = 5.70, *P* = 0.049) and no other main effects or interactions (all *P* > 0.09). Anodal tDCS reduced handwriting APP in both groups (FHD pre 488.97 ± 77, post 426.31 ± 42; control pre 498.15 ± 75, post 434.35 ± 65), while cathodal tDCS reduced handwriting APP in the control group only (pre 525.69 ± 87, post 433.67 ± 62) (Figure 3D). For the fast cyclic drawing task there was a main effect of TIME (*F*_1,7_ = 11.45, *P* = 0.012) and a CONDITION by TIME interaction (*F*_2,14_ = 6.79, *P* = 0.013), but no other main effects or interactions (all *P* > 0.19). There was a reduction in APP during fast cyclic drawing in the control group (pre 820.34 ± 60, post 619.25 ± 58, *P* = 0.042) and a trend for a reduction in the dystonia group (pre 759.86 ± 55, post 599.98 ± 57, *P* = 0.057) only after anodal tDCS (Figure 3E). There was no effect of cerebellar tDCS on APP during the light cyclic drawing task (all *P* > 0.21, Figure 3F).

## Discussion

Contemporary neuro-rehabilitation supports the translation of findings from basic science to humans in the context of promoting brain recovery in neurological disorders. Recent findings in dystonic animal models implicate the cerebellum in the pathophysiology of focal dystonia (Wilson and Hess, 2013). Therefore, there may be novel opportunities to improve function in FHD by modulating the output from the cerebellum using non-invasive techniques. In the current study we examined the effect of anodal, cathodal and sham tDCS to the cerebellum on handwriting and circle drawing kinematics, and corticomotor excitability and CBI in people with FHD and controls. There was a negative correlation between CBI and handwriting assessed by the WCRS, in that more CBI was associated with higher (worse) WCRS scores. There was no difference between cortical neurophysiology in FHD and control participants at baseline. However mean stroke frequency was lower during handwriting and fast cyclic drawing, and average pen pressure was higher during light cyclic drawing, in the dystonia group. There was an effect of cerebellar tDCS on cortical neurophysiology where anodal tDCS reduced CBI to the same degree in both groups. Anodal tDCS of the cerebellum modified mean stroke frequency and reduced average pen pressure during handwriting and fast cyclic drawing, indicating there may be potential for cerebellar tDCS to provide a novel treatment intervention in FHD. There was no correlation between the effect of cerebellar anodal tDCS on the change in CBI and any kinematic measure for either group, indicating the neurophysiological mechanism underlying kinematic improvements has not yet been elucidated. The results of this pilot study have potential implications for the rehabilitation of FHD, supporting this translational approach to identifying a novel and effective intervention.

We found kinematic parameters during handwriting and cyclic drawing could differentiate between dystonia and control subjects at baseline, in agreement with previous studies (Zeuner et al., 2007; Schabrun et al., 2009). The finding that cerebellar anodal tDCS modulated mean stroke frequency and average pen pressure during handwriting extends those of a previous study where handwriting was unchanged by cerebellar anodal tDCS, assessed with the WCRS and investigator or self-rated assessment of handwriting speed (Sadnicka et al., 2014). Interestingly, we showed mean stroke frequency and average pen pressure was reduced during handwriting after stimulation. This is a dichotomous finding, as slower handwriting could signify a worsening of symptoms, while reduced pen pressure during writing indicates an improvement in dystonic hand function. It is unclear why this was the case in the current study. Handwriting did not appear degraded during performance of the task; in fact most of the dystonia group reported greater ease of handwriting following anodal stimulation. Since average speed increased and pressure decreased during the fast cyclic drawing task, we consider our overall findings indicate the effects of anodal tDCS were positive for improving hand function. Light cyclic drawing demonstrated similar trends as fast cyclic drawing following stimulation, but due to variation in task performance amongst the dystonia group and the small numbers of participants, did not reach statistical significance. It is interesting that cathodal tDCS appeared to evoke similar responses to anodal tDCS for handwriting but not cyclic drawing. While it is unclear why anodal and cathodal cerebellar tDCS can have similar effects, the finding is consistent with previous reports for both neurophysiology and behavioral measures (Ferrucci et al., 2013; Sadnicka et al., 2014). Even though the current results are exploratory and underpowered to show significant effects, effects on dystonic hand function are promising and larger studies using repeated sessions of cerebellar anodal tDCS, with longer post stimulation periods are warranted.

Our second finding was that CBI was present in people with focal hand dystonia. This result is at odds with a previous study where CBI was not observed in people with FHD (Brighina et al., 2009), but agrees with another where CBI was present in people with cervical dystonia (Koch et al., 2014). The disparity between our study and findings of Brighina et al. (2009) might be explained by technical differences in the method used to evoke CBI. We used a flat figure of eight coil rather than a cone coil, and also determined the intensity used for conditioning the cerebellum using a different method. It may also be possible that CBI is variable and dependent on the severity of dystonia, so studies using patients with different severity levels may generate disparate results. We did find that greater CBI was associated with worse hand function, as assessed by the WCRS, prior to stimulation in the hand dystonia group. A future study comparing CBI between dystonia participants stratified for severity based on the WCRS might help to answer this question. Because we expected to see a reduction in CBI, our *a priori* hypothesis was that cerebellar tDCS would increase (normalise) CBI. However, we found CBI was reduced following stimulation which agrees with previous studies demonstrating a reduction in CBI following intermittent TBS applied to the cerebellum in cerebellar stroke (Bonnì et al., 2014) and in cervical dystonia (Koch et al., 2014). However, in those studies changes in CBI were accompanied by clinical improvements, which we did not observe. This indicates that CBI is unlikely to be the key neurophysiological mechanism responsible for focal hand dystonia. The improvements in hand function from cerebellar stimulation may result from modification of alternate output projections such those as to brainstem nuclei (Bradnam and Barry, 2013), which could be explored in future studies.

## Study Limitations

The major limitation of this study is the small number of participants, meaning it was underpowered to show strong clinical effects. However, as an exploratory study, it has revealed an important potential for cerebellar tDCS as a novel treatment intervention for FHD. A limitation of our neurophysiological data collection method is that we used a single conditioning stimulus intensity to evoke CBI in both dystonia and control groups. A difference in the intensity of conditioning stimulus required to evoke CBI in healthy individuals was noted recently (Baarbé et al., 2014). It might be that a conditioning stimulus-response curve would provide better information regarding the optimum intensity to evoke CBI in individuals. Such an approach would elucidate whether deficits in CBI result from higher CBI thresholds (relative to RMT of M1) in patients compared to controls (Baarbé et al., 2014). Another limitation is that we used a flat figure of 8 coil over the cerebellum to evoke CBI, which produces less CBI than with deeper stimulation cone coils (Hardwick et al., 2014). However, the degree of inhibition we observed (around 20%) is similar to that described in healthy controls and people with ataxia following cerebellar stroke, also using a flat figure of 8 coil (Bonnì et al., 2014; Brusa et al., 2014). Finally, we assessed CBI in a hand muscle at rest. CBI is modulated at the onset of a muscle contraction (Kassavetis et al., 2011) and other neurophysiological measures in people with upper limb dystonia have been found only at movement onset and not at rest (Gilio et al., 2003; Weise et al., 2012). A future study should test CBI in both resting and at onset conditions.

## Conclusions

In a translational approach to neuro-rehabilitation we applied the growing body of basic neuroscience research that cerebellar dysfunction contributes to the pathophysiology of dystonia (Standaert, 2011; Sadnicka et al., 2012; Filip et al., 2013; Wilson and Hess, 2013) in an exploratory study in humans using cerebellar non-invasive stimulation. Our results suggest anodal cerebellar stimulation may have benefits for dystonic hand function although further studies using larger numbers of participants are needed to confirm these findings. However, kinematic assessment of handwriting and cyclic drawing tasks hold promise as outcome measures in studies investigating novel interventions for focal hand dystonia.

## Conflict of Interest Statement

The authors declare that the research was conducted in the absence of any commercial or financial relationships that could be construed as a potential conflict of interest.
